# Lipid mediators in immune dysfunction after severe inflammation^☆^

**DOI:** 10.1016/j.it.2013.10.008

**Published:** 2014-01

**Authors:** James N. Fullerton, Alastair J. O’Brien, Derek W. Gilroy

**Affiliations:** Centre for Clinical Pharmacology, Division of Medicine, Rayne Institute, 5 University Street, University College London, London, WC1E 6JF, UK

**Keywords:** eicosanoids, prostaglandins, cyclooxygenase, resolution of inflammation, sepsis, immunosuppression

## Abstract

Sepsis, trauma, burns, and major surgical procedures activate common systemic inflammatory pathways. Nosocomial infection, organ failure, and mortality in this patient population are associated with a quantitatively different reprioritization of the circulating leukocyte transcriptome to the initial inflammatory insult, greater in both magnitude and duration, and secondary to multiple observed defects in innate and adaptive immune function. Dysregulation of inflammatory resolution processes and associated bioactive lipid mediators (LMs) mechanistically contribute to this phenotype. Recent data indicate the potential efficacy of therapeutic interventions that either reduce immunosuppressive prostaglandins (PGs) or increase specialized proresolving LMs. Here, we reassess the potential for pharmacological manipulation of these LMs as therapeutic approaches for the treatment of critical illness (CI).

## Highlights

• Aberrant LM levels contribute to immune dysfunction in CI.
• Aberrance reflects dysregulation of inflammatory resolution pathways or their failure.
• Targeted manipulation of LMs restores immune competence and outcomes in animal models.
• Stratified resolution-based immunomodulatory strategies hold therapeutic potential.

## Inflammation unites CI

Systemic inflammation is nearly ubiquitous in CI, uniting the leading causes of intensive care (ICU) admission [1]. Induced by infectious and noninfectious stimuli with approximately equal frequency [2], the host inflammatory reaction is driven by common mediators and shared signaling pathways 3, 4. Critically ill patients are now understood to experience a highly coordinated, reproducible response at the transcriptomic, metabolomic, and proteomic level, regardless of the inflammatory source 5, 6, 7.

Adverse clinical outcomes are associated with a quantitative dysregulation of the inflammatory profile in both magnitude and duration [7]. Predominance and prolongation of anti-inflammatory processes mechanistically contribute to multiple defects in the innate and adaptive immune system, and consequent vulnerability to nosocomial [hospital-acquired infection (HAI)] 8, 9 and CI-induced immune dysfunction (CIIID) [10]. In total, ∼30% of CI patients will contract HAI; a rate six times greater than on standard wards 11, 12. HAI in this setting is associated with a twofold greater inpatient mortality risk and a case mortality in excess of 50% 11, 13. Causative pathogens are most commonly bacterial; however, fungal infections (particularly *Candida* spp.) are increasing in incidence [12]. Viral infection, especially reactivation, poses an additional risk, and polymicrobial infection is common.

LMs, including eicosanoids and the more recently discovered ‘specialized pro-resolution lipid mediators’ (SPMs), are key signaling molecules in the resolution of inflammation, playing a pivotal role in regulating the inflammatory profile and promoting return to homeostasis [14]. Their dysregulation in any of several dimensions may have pathogenic consequences (Box 1), with failure of resolution leading to chronic inflammation and excess tissue damage being best recognized 14, 15. The potential active contribution of LMs to an anti-inflammatory, immunosuppressive phenotype has, until recently, received little attention [10].

Box 1 **LMs: background and pathogenic potential**

AA (20:6, ω-6), docosahexaenoic acid (22:6, ω-3) and eicosapentaenoic acid (20:5, ω-3) are polyunsaturated fatty acids that form the substrates for the enzymatic generation of several groups of bioactive LMs. Eicosanoids – the generic term for a group of bioactive lipids containing 20 carbons derived from AA – are divided into several separate mediator families, the major groups being the PGs, LTs and LXs. More recently discovered ω-3-derived SPM families include Rvs, protectins, and maresins. LMs represent vital endogenous biochemical determinants of inflammatory kinetics and the principle mediators of resolution.

The ability of NSAIDs to reduce the primary symptoms and signs of inflammation via COX inhibition and hence PG suppression has led to a common assumption that they, and in turn eicosanoids in general, are universally proinflammatory. This represents a grossly simplified view. These lipid mediators are variably constitutive and inducible, expressed widely yet in a cell-type- and tissue-specific manner, and their actions are diverse, multifaceted, and vary down to the receptor level. Individual molecules have been shown to variably exert pro- and anti-inflammatory effects along with pro-resolution properties in a context-dependent manner.

Given their immunomodulatory potency and diversity of action, for an effective and self-limited inflammatory reaction to be facilitated, eicosanoid generation must be localized, balanced, proportionate, and timely. Disturbance in any of these dimensions in isolation, or more likely combination, may contribute adversely to disease states. Several pathogenic aberrations may be hypothesized:

1) Location of action.
  • Compartment leakage or altered distribution of generation. Endocrine as opposed to typical autocrine or paracrine activity.
2) Increased or decreased concentration.
  • Altered synthesis, via host, pathogen or iatrogenic intervention, through modulation of substrate or enzymatic process.
  • Promotion or loss of catabolism (local or systemic) or the failure of feedback loops. Altered bioavailability or protein binding (e.g., albumin).
3) Deranged temporal profile of production.
  • Failure or dysregulation of eicosanoid class switching 64, 65.
4) Up- or downregulation of receptors, alteration in receptor profile, including distribution.
5) Modification of action (e.g., co-stimulation – additive, synergistic, or anergic) by other stimuli/mediators in the surrounding inflammatory milieu.

In broad terms, two key patterns may result from the above. First, deficient or failed resolution where either an insufficient concentration of LM are available to facilitate inflammatory termination, or their action is inadequate. This has been recently discussed in varying inflammatory conditions [14]. Second, a state we describe as injurious resolution may exist. Here, an excessive immunoregulatory effect is exerted by eicosanoids involved in the initiation or control of resolution, rendering host defenses locally or systemically compromised [10].

Observational studies have related LM-modifying aspirin and statin administration, as well as nonsteroidal anti-inflammatory drugs (NSAIDs), to clinical benefit in the CI population (Box 2) [16], and compelling clinical data support the use of targeted immunomodulatory therapy 17, 18. This information, coupled with a more advanced appreciation of how inflammatory resolution pathways, and LMs in particular, may affect immune competence (and be used to modify it) in CI, necessitates a reappraisal of the clinical validity of these drugs.

Box 2 **Observational and mechanistic data supporting therapeutic LM manipulation**

Regular use of aspirin in patients who develop community-acquired pneumonia has been associated with lower ICU requirement (odds ratio 0.19, 95% confidence interval 0.04–0.87) and shorter in-patient stay (13.9±6.2 vs. 18.2±10.2 days) [86]. In a general CI patient set prior prescription and consumption of aspirin and statins has been linked with reduced severity of illness (development of severe sepsis, acute lung injury, or adult respiratory distress syndrome) and mortality in a multivariate analysis [83]. Aspirin administration within 24 h of SIRS recognition has been separately linked with a significant decrease in mortality in all such ICU patients of –6.2% (absolute risk difference after propensity matching), and an even greater mortality reduction in those with proven sepsis of –14.8% (27.4% aspirin vs. 42.2% no aspirin) [84]. Independently, a near 50% reduction in the risk of inpatient mortality in septic patients given aspirin during their ICU admission has been described [88], with similar levels of benefits potentially resulting from utilization of alternative NSAIDs in addition (ibuprofen, diclofenac, or indomethacin) [85]. NSAID benefit is however lost if coadministered with aspirin [85], and concerns regarding delayed presentation [89] and side effects persist [84]. The cause of clinical improvement is likely multifactorial and suggested to be secondary to antithrombotic (antiplatelet), anti-inflammatory effects and augmentation of inflammatory resolution pathways 16, 87. This is re-enforced via positive interactions with statins [83] and other antiplatelet agents 86, 87.

Mechanistically, aspirin and statins exert their immunomodulatory actions through increased synthesis of bioactive SPMs from all three PUFAs (Figure 3). Both may modify COX-2 – aspirin via serine acetylation, and statins via cysteine *S*-nitrosylation – to generate 15*R*-hydroxyeicosatetraenoic acid, which is subsequently converted by 5-LOX into 15-epi-LXA_4_ (aspirin-triggered LX, ATL). Aspirin may in addition upregulate expression of the ATL receptor ALX (FPRL1) [42], and promote the generation of aspirin-triggered Rvs (17*R*-epimers) and protectins (e.g., aspirin-triggered protectin D1). Glucocorticoids have also been demonstrated to increase SPM generation [79]. The effects of these molecules in models of CI are discussed in the main text.

Here, we summarize contemporary preclinical and clinical data describing the effects of modulating LMs in CI syndromes, predominantly sepsis. We describe the pathogenic contribution of two distinct yet interlinked patterns of dysregulation to CIIID, namely injurious and failed inflammatory resolution, and discuss therapeutic opportunities presented by LM manipulation.

## CI: a failure of resolution?

ω-3-Derived SPMs from different series appear to have individually separate yet collectively beneficial effects on multiple modalities of immune function. Evidence indicates that a paucity of these LMs contributes to derangement of the inflammatory profile and CIIID, with therapeutic replacement restoring or augmenting immune function. In the next sections we discuss data relating to specific LMs of the resolvin (Rv) and protectin series, and later the lipoxin (LX) and leukotriene (LT) families in interventional animal models of infection/inflammation.

Defining features of SPM bioaction include the ability to: (i) counter-regulate mediators that summon leukocytes, in particular polymorphonuclear cells (PMNs, neutrophils), to an inflamed site; (ii) dampen pain; (iii) stimulate nonphlogistic monocyte recruitment; and (iv) activate macrophages to efferocytose apoptotic granulocytes and clear both pathogens and tissue debris [14]. Despite being part of the endogenous anti-inflammatory process via action (i), with associated prevention of inflammatory amplification, it is attributes (iii) and (iv) in tandem with promotion of phagocyte trafficking to lymph nodes [19] that distinguishes them from classical anti-inflammatory mediators such as interleukin (IL)-10 or IL-1 receptor antagonist [14].

SPMs have repeatedly been demonstrated to lack an immunosuppressive action, and indeed to augment host-directed antimicrobial defenses [20]. These molecules stimulate mucosal production of bactericidal peptides [21] and enhance bacterial phagocytosis by PMNs and macrophages, working synergistically with antibiotics, to increase their therapeutic action and hence bacterial clearance [22]. They have further been shown to suppress nuclear viral mRNA transcript export, and hence replication, reducing mortality from influenza infection [23]: a potentially novel therapeutic addition to standard antivirals, focused on modifying host immune capability, avoiding the problems posed by these infectious agents diversity, variability and capacity to evolve.

### Rvs and protectins

RvE1 administered prior to a murine model of aspiration pneumonia (hydrochloric acid with subsequent *Escherichia coli* challenge) was associated with a reduction in proinflammatory cytokines, decreased pulmonary PMN accumulation, enhanced bacterial clearance, and improved survival [24]. El Kebir and colleagues have further described the ability of RvE1 to promote resolution of established infective and sterile models of murine lung injury [25]. Mechanistically, RvE1 was noted to enhance NADPH-oxidase reactive oxygen species generation and promote phagocytosis-induced neutrophil apoptosis (with subsequent efferocytosis by macrophages) via the LTB_4_ receptor BLT1. Increased activation of caspase-8 and caspase-3 in tandem with attenuation of both extracellular signal-regulated kinase (ERK) and Akt-mediated apoptosis-suppressing signals shifting the balance of pro-/anti-survival information toward apoptosis via induction of mitochondrial dysfunction. In addition, RvE1, at concentrations as low as 1 nM, enhances macrophage phagocytosis, with the products of its metabolism continuing to exert pro-resolution properties but with reduced bioactivity *in vivo* [26].

RvD1 pretreatment prior to lipopolysaccharide (LPS)-induced acute lung injury is protective, improving pathological changes and survival [27]. The central mechanism appears to be suppression of nuclear factor (NF)-κB activation in a partly peroxisome proliferator-activated receptor (PPAR)γ-dependent manner, with associated reduction in downstream signaling/transcriptomic alteration [28]. RvD2, but not its isomer *trans-*RvD2, has been shown specifically to improve survival in murine polymicrobial sepsis (cecal-ligation and puncture; CLP). Its actions appear multifaceted – modulating leukocyte–endothelium interactions in a direct (adhesion receptor expression) and indirect manner (endothelial NO production), altering the cytokine profile [reduced IL-17, IL-10, PGE_2_ and LTB_4_], and enhancing bacterial phagocytosis and intraphysosomal vacuolar production of reactive oxygen species [20]. More recently, the ability of RvD2 to restore neutrophil directionality, prevent CIIID, and thus increase survival from a secondary septic challenge post-burn injury has been demonstrated [29].

Discrete specialized pro-resolution mediators are unlikely to be produced in isolation and have overlapping proresolving actions. RvE1, aspirin-triggered (ATL, 15-epi-lipoxin A_4_) and protectin D1 may independently rescue cyclo-oxygenase (COX)- and lipoxygenase (LOX)-derived ‘resolution deficits’ *in vitro* and *in vivo*, with actions extending to promotion of phagocyte trafficking away from the primary inflammatory site [19]. The ability to bind and act as agonists on alternate SPM receptors (e.g., RvD1 on the LXA_4_ receptor [27]) may provide one pharmacological explanation for this phenomenon. However, despite their common actions the source of different classes of SPMs in inflammation appears diverse. Recent evidence suggests that RvE1 and 2 are synthesized by PMNs via the 5-LOX pathway [30], whereas eosinophils are responsible for generation of 12/15-LOX-derived mediators protectin D1 and the newly discovered RvE3 31, 32. Deficiency of these cell types in the resolution phase may lead to impaired biosynthesis with deleterious consequences [31]. The same may be true of polyunsaturated fatty acids at the inflammatory site.

Experimentally, the ω-3 Rv precursors eicosapentaenoic and docosahexaenoic acids have been demonstrated to increase in exudates during the resolution phase; being both plasma (partially bound to leaked albumin) and locally derived 14, 33. Indirect evidence to support the therapeutic benefit of increasing this SPM series concentration in humans comes from the addition of fish oils to parenteral nutrition in septic patients. A raised plasma eicosapentaenoic concentration was observed along with modification of the cytokine profile, and small physiological and clinical benefits in a recent randomized clinical trial [34].

### LTs and LXs

Therapeutic use of the arachidonic acid (AA)-derived LX series may also be beneficial. Post-insult treatment with LXA_4_ has been demonstrated to limit inhaled LPS-induced lung injury [35], and to reduce pro- and anti-inflammatory cytokine production, enhance macrophage recruitment, reduce blood bacterial load, and improve mortality in a rat CLP model [36]. In this later study, macrophage recruitment was increased without impairing phagocytic function, and systemic inflammation reduced without increasing bacterial spread, mirroring the previously described observations with other SPMs 20, 24.

A similar paradoxical relation between an attenuated innate immune response (PMN trafficking to the infected site), yet efficacy of the overall inflammatory process, as determined by survival, has been demonstrated in both wild type mice treated with MK 886 (a 5-LOX inhibitor) and in 5-LOX-deficient mice [37]. This effect could be partially replicated if antagonists of cysteinyl-LTs (a family including LTC–F_4_) were given, but crucially not with antagonism of the classically proinflammatory LTB_4_. This elegantly demonstrates the hierarchical, multifaceted and often opposing effects of eicosanoids in sepsis. In this setting it appears that the prevention of the cysteinyl-LTs deleterious effects on the vasculature, hence host hemodynamics, assumes primacy as the main cause of benefit in 5-LOX antagonism. By contrast, selective LTB_4_ inhibition prior to and post-CLP appears to have little effect on vascular tone and permeability, but may blunt the innate immune response – specifically neutrophil trafficking – exacerbating the infective insult 38, 39.

The complex interplay between AA-derived LMs in sepsis has also been highlighted in recent data showing that flavocoxid, a dual COX-2 and 5-LOX inhibitor, reduces the expression of NF-κB, COX-2, and 5-LOX with improved survival in a murine CLP model [40]. Plasma IL-10 and LXA_4_ concentrations were increased while tumor necrosis factor (TNF)-α, IL-6, LTB_4_, and PGE_2_ were decreased. Whether the observed improvement in outcome was due to enhanced pro-resolution effects driven by increased LXA_4_, decreased cytokine storm (TNF-α and IL-6), augmentation of the immune response via reducing PGE_2_ and 5-LOX derived LTs (discussed below), selective shunting of AA down the COX or LOX pathways, or a combination of the above is unclear.

Separately, biologically active ATL and aspirin-triggered Rvs have been noted to have pro-resolution effects, inhibiting leukocyte trafficking in a NO-dependent manner in both murine [41] and human [42] inflammation, and downregulating superoxide production in neutrophils along with macrophage inflammatory peptide 2 and IL-1β production [43]. The importance of ATL and LXA_4_ inhibitory stimulation has been previously demonstrated in CI; its absence leads to unbridled inflammation and elevated mortality in animal models of infection due to dendritic cell (DC) hypersensitivity [44].

**Figure 1:**
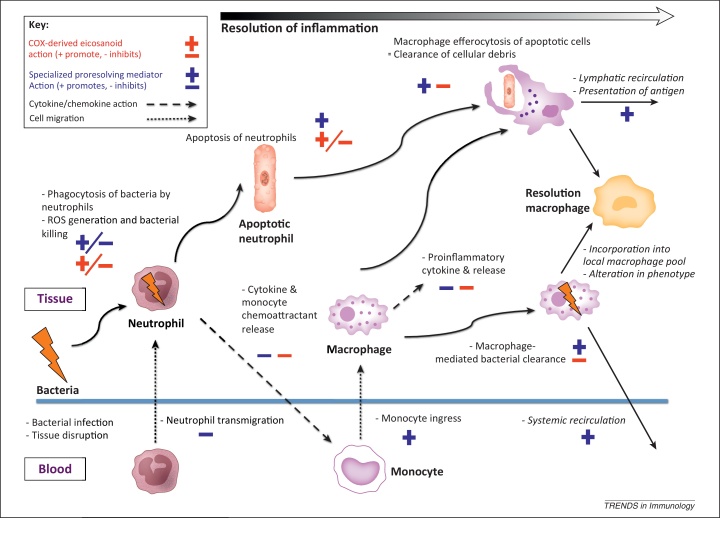
Immunomodulatory effects of two broad classes of lipid mediators throughout the inflammatory response. Persistence or excessive levels of cyclooxygenase-derived prostanoids and leukotrienes due to a failure to class-switch may contribute to injurious resolution. Here, mediators designed to promote inflammatory resolution multimodally drive an immunosuppressive, anti-inflammatory phenotype leading to susceptibility to secondary nosocomial infections (highlighted in red). Predominantly ω-3 polyunsaturated fatty acid lipoxygenase-derived specialized proresolving lipid mediators nonphlogistically augment key processes in inflammatory resolution. An absence or deficit of these eicosanoids may lead to chronic inflammation, failure of pathogen clearance, and both local or distal tissue damage.

Thus, accumulating evidence supports the notion that SPMs are both necessary to the host immune response and beneficial in severe inflammatory states. Increasing LX, Rv, and protectin concentrations augments host defense and improves survival in preclinical models of CI via multimodally enhancing innate immune function and ameliorating CIIID. The immunomodulatory actions of SPMs on inflammatory resolution processes are summarized in Figure 1.

## Contribution of excessive, injurious resolution pathways

The majority of the interventional studies described above have used a paradigm of substrate supplementation, direct molecule addition, or aspirin-augmented biosynthesis to highlight the benefits of SPMs in CI. Their pathogenic contribution to severe inflammatory states thus appears to be one of absence or insufficiency. Conversely, excess (absolute or relative) or persistence of early-phase resolution-regulating LMs, specifically prostanoids, may be equally deleterious to host immune function: a state of ‘injurious resolution’ [10]. The negative immunomodulatory effects of PGE_2_ [45], PGD_2_ [46], and the cyclopentenone PGs of the J_2_ series [47] have recently been reviewed and are not described in detail here. Indications of their role in CI is consequently derived from the reverse methodology – their reduction or antagonism. Specifically ‘two-hit’ inflammatory/infective insult models may be of particular relevance to studying CIIID [48].

Severe inflammatory stress in mice is associated with a predominantly anti-inflammatory state and relative immunosuppression following the initial proinflammatory response. This is characterized by increased susceptibility to and worse outcomes from infection 48, 49, 50, mimicking clinical observations [51]. Vulnerability to secondary infective challenge (replicating HAI) is dependent on time post-initial insult, decreasing with increased temporal separation 49, 50, and may be reduced by therapeutic strategies designed to restore immune function [49]. PGE_2_ appears to be a key mediator of this phenomenon. Bronchoalveolar lavage fluid in mice subjected to pulmonary *Aspergillus fumigatus* conidia challenge post-CLP contains higher amounts of PGE_2_, the production of which seems dependent upon both alveolar macrophages and epithelial cells [52]. Treatment with ketoprofen, a nonselective COX-inhibitor, after CLP, but prior to fungal challenge, reduced the PGE_2_ concentration by >95%, enhanced neutrophil recruitment, macrophage phagocytosis and proinflammatory cytokine secretion, leading to a fourfold increase in survival. The E-prostanoid (EP)4 receptor in particular was described as playing a pivotal role [52].

These data support previous work also demonstrating the efficacy of COX-2 inhibition (NS-398) in attenuating prolonged, excessive PGE_2_ production secondary to an alternate severe inflammatory stressor (trauma/hemorrhage), with resultant reduction in mortality from subsequent infective challenge 53, 54. In addition to EP4, a PGE_2_–EP2–cAMP axis also appears to contribute to reduced effector cell function, with both specific EP2 receptor antagonists or EP2-deficient mice preventing PGE_2_-induced impairment of both Fcγ-receptor-dependent phagocytosis and NADPH-oxidase-mediated bacterial killing 55, 56.

The temporally defined window of immunosuppression is matched by sequential alterations in COX-2 expression and PGE_2_ synthesis. Higher circulating plasma concentrations of PGE_2_ are observed on both Days 1 and 7 post-severe inflammatory stress compared to controls, indicating exaggerated and prolonged prostanoid production [54]. Correspondingly, splenic macrophages have increased COX-2 mRNA induction in response to LPS stimulation even at Day 7 53, 54. Increased PGE_2_ concentrations may alternatively stem from selective induction of microsomal PGE_2_ synthase (mPGES)-1 by CI states independent of COX-2 expression [52]. In comparison, peritoneal neutrophils from burn-injured animals have been reported to exhibit a late-phase decrease in COX-2 expression and PGE_2_ synthesis, coupled with a lack of induction to secondary infective challenge [57], likely indicating compartmentalization of the inflammatory response 58, 59.

AA release and its COX-mediated metabolism are also modified by CI in humans. In patients with fracture or burn injury, peripheral blood mononuclear cells exhibit increased COX-2 mRNA and PGE_2_ synthesis in response to LPS stimulation [60]. By contrast, COX-2 gene expression in septic patients is reduced such that both basal circulating concentrations of its metabolites (including PGE_2_), and those induced by LPS stimulation of blood leukocytes *ex vivo*, were lower than in healthy controls [61]. Furthermore, the degree of AA-metabolism derangement was associated with disease severity (greater in septic shock vs. severe sepsis), and failure of its recovery between admission to ICU and Day 3 post-admission was predictive of adverse clinical outcome (prolonged admission or death). The authors speculate that reduced prostanoid generation forms part of the anti-inflammatory CIIID phenotype. In attempting to reconcile these data, we propose two alternative but interlinked explanations: (i) that altered AA metabolism and PGE_2_ generation in the blood compartment represents an adaptive change to prevent systemic inflammation 58, 62; or (ii) a response to excess production extravascularly (i.e., the primary infective/inflammatory site). Up- and downregulation of prostanoid receptors has been observed in CI humans [60], along with alterations in COX-2 and mPGES-1 expression [61], indicating a dynamic, responsive system. Late-phase immunosuppressive mediators are thought to arise from tissue-resident macrophages *in vivo* [63]. Chronic release into the circulation and endocrine action initiated by the primary event may be expected to induce compensatory adaptions in blood leukocytes.

The above data indicate that, contrary to popular conceptions, COX-derived eicosanoids, and in particular PGE_2_, have significant anti-inflammatory and immunosuppressive effects. In both animal and human studies, CI induces alteration of prostanoid production. Sustained or excessive production of these resolution-regulating eicosanoids may be pathogenic through increasing susceptibility to secondary infection. The efficacy displayed by COX inhibition in clinical observational studies and either COX inhibition or PGE_2_ antagonism in preclinical work warrants further investigation. The immunomodulatory actions of COX-derived eicosanoids are summarized in Figure 1.

## A single or dual defect?

It is unlikely that either an excess of immunosuppressive eicosanoids or a deficiency of specialized pro-resolution mediators exists independently in CI; their production being inextricably interlinked. Over the course of the inflammatory response, LM profiles undergo a ‘class switch’ from initial-phase COX-derived PGs and LTs to specialized pro-resolution mediators of the LX, Rv and protectin series, with PGE_2_ playing a controlling role 64, 65. In particular, there is now evidence to suggest that different phagocytic cell types and their subpopulations display specific eicosanoid profile signatures that are dynamically altered at defined intervals throughout inflammation, influenced by the surrounding milieu and ingested material [66].

**Figure 2:**
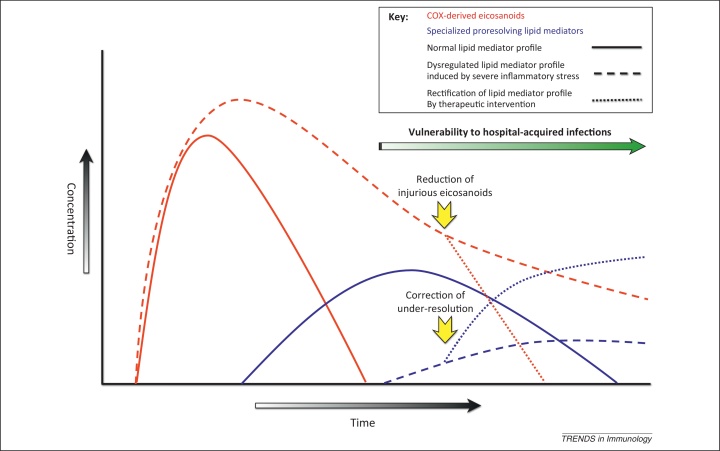
Proposed dysregulation of resolution-phase lipid mediators in critical illness. Solid lines indicate the normal inflammatory profile, with an early rise in cyclooxygenase (COX)-derived prostaglandins (PGs) and 5-lipoxygenase-derived leukotriene B_4_, which trigger a subsequent rise in specialized proresolving lipid mediators (SPMs) including lipoxins, resolvins, and protectins. Dashed lines display the altered profile of eicosanoids in critical illness-induced immune dysfunction, with persistence of early-phase lipid mediators that exert negative immunomodulatory effects and a paucity or relative insufficiency of SPMs, which nonphlogistically augment several key resolution pathways including bacterial clearance. The combination of this dual defect contributes to vulnerability to hospital-acquired infection. Identification of aberrant eicosanoid profiles in critically ill patients may allow their therapeutic correction or antagonism to ameliorate effector cell functional impairment as indicated by the dotted lines.

Recent data demonstrate that severe inflammatory stress undermines these normally tightly regulated resolution programs. In a murine peritonitis model contrasting low-dose, self-resolving inflammation with high-dose, nonresolving inflammation (10 mg vs. 1 mg zymosan), sustained high amounts of PGE_2_ and LTB_4_ (>5× normal) were reported in the high-dose exudates, along with persistently compromised specialized pro-resolution mediator production (LXA_4_, protectin D1 and Rvs ∼3-fold less than normal concentrations) [67]. Alterations in miRNA expression, specifically miR-219-2, and subsequent target gene expression were implicated. This situation appears analogous to the dysregulated transcriptomic and inflammatory response associated with adverse outcomes in CI [7]. Humans display different inflammatory response profiles to set stimuli that are largely determined by resolution processes and mediators, broad categories of ‘resolution phenotype’ being established preclinically [68], and tentatively in various clinical settings 69, 70. We speculate that inflammatory stress of sufficient magnitude in individuals rendered susceptible via their resolution phenotype may trigger both higher and persistent amounts of initial-phase eicosanoids with concomitant failure of specialized pro-resolution mediator generation, leading to both injurious and failed resolution (Figure 2). Such dysregulation of LM synthesis may both exacerbate the acute systemic inflammatory response syndrome (SIRS) and subsequently contribute mechanistically to late-phase CIIID [10].

## Harnessing therapeutic opportunities

**Figure 3:**
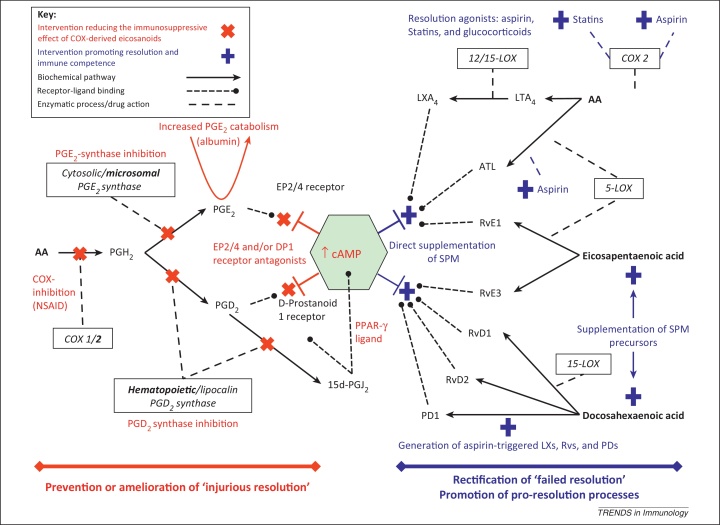
Potential therapeutic interventions to modify resolution defects and improve innate effector cell functionality in critical illness. Targets on the left of the diagram (red) describe means of reducing excessive or prolonged production of immunosuppressive COX-generated PGs or antagonizing their action. Targets on the right (blue) indicate means of supplementing and/or augmenting levels of SPMs that nonphlogistically enhance multiple effector modalities. AA, arachidonic acid; ATL, aspirin-triggered lipoxin (15-epi-LXA_4_); COX, cyclooxygenase; EP2/4, E prostanoid 2/4 receptor; LOX, lipoxygenase; LT, leukotriene; LX, lipoxin; PD, protectin; PG, prostaglandin; PPAR, peroxisome proliferator-activated receptor; Rv, resolvin; SPM, specialized proresolving lipid mediator.

The recognition of aberrant inflammatory resolution pathways as a contributory factor to the CI clinical phenotype opens several translational therapeutic avenues (Figure 2, Figure 3).

The first derives from the principle of non-maleficence: cessation of, or alteration in, the use of existing therapies and/or practices that impair the resolution phase, or cause its dysregulation. Candidate drugs may be identified via resolution indices (for example [19]) in standardized models. Widely used drugs in anesthetics and critical care have already been implicated [71], and the use of alternate agents or the same drugs at appropriate phases of inflammation may be advocated. As an example, NSAIDs may adversely blunt the initial inflammatory response through impairment of proinflammatory mediator generation, but may hypothetically be used to ameliorate the late immunosuppressive contribution of COX-derived prostanoids, especially PGE_2_, to CIIID 52, 53, 54, 72.

The potential for both immunostimulatory and immunosuppressive agents in the management of CI in general, and sepsis in particular, is well recognized and multiple potential targets have been identified (see [73] for recent review). Real-time immunological assays to determine host immune competence and guide immunomodulatory therapeutics have already shown promise in both experimental [74] and clinical 17, 18 studies. As already discussed, interindividual variance in inflammatory response to a given stimulus may partly be attributed to expression of pro-resolution processes [68]. Eicosanoid serum concentrations, *in-vitro/ex-vivo* functional assays and flow cytometry may be used to stratify patients into those who will benefit from targeted interventions to correct resolution aberrancies: a form of individualized medicine [75]. Evidence that such a strategy may be effective is already available: a subgroup of patients in the major randomized controlled trial of ibuprofen in sepsis who exhibited exaggerated prostanoid production displayed reduced mortality secondary to COX inhibition (Box 3) [76].

Box 3 **History of COX inhibition in CI**

The concept of using COX inhibition in the setting of CI – predominantly sepsis – is not new; the first experiments being performed in the 1960s utilizing aspirin. Since then a vast amount of data has accumulated. Although animal work has been overwhelmingly positive, COX inhibition pre- or post-septic challenge improving survival in >70% (31/43) of publications and being detrimental in only 6.9% (3/43) [72], this success has not been replicated in humans. The largest randomized controlled trial showed no significant improvement in sepsis-related mortality (37% ibuprofen vs. 40% placebo) despite demonstrable prostanoid suppression and improvement in physiological variables [90]. Two further smaller studies also showed no effect on mortality (ibuprofen [91] and lornoxicam [92]). All trials have however supported the safety of NSAID use in CI and fears that COX inhibition via aspirin or NSAIDs may predispose to severe sepsis have not been substantiated [89].

Why then has this preclinical promise not been fulfilled? Many of the standard difficulties of ICU and sepsis trials apply to these studies. Investigated populations were heterogeneous in age, infection site, causative pathogen, and comorbidity burden, producing a high noise-to-signal ratio. Enrollment over a range of time-points post acquirement of infection was inevitable, preventing standardized intervention in the same phase of disease and potentially missing a narrow therapeutic window where adverse outcomes may be averted. Trial design was imperfect, the major randomized controlled trial being underpowered, aiming to detect an unrealistic 35% difference in mortality [16]. Most importantly, we now understand that the rationale behind the therapeutic strategy may have been flawed. NSAID therapy was directed at the proinflammatory phase, aiming to ameliorate SIRS not CIIID, and given to a diverse patient set in a nonstratified manner [73].

Re-evaluation of the previously described publications supports this view. A greater beneficial therapeutic effect (survival) of NSAIDs was observed in animal models involving live pathogens than in those where pathogen-associated molecular patterns (PAMPs; e.g., LPS) were administered alone [72]. This differential and enhanced efficacy may be explained by suppression of immunosuppressive eicosanoids and consequent augmentation of innate immune function, as opposed to abrogation of their negative physiological effects, which would be equivalent in both live pathogen and PAMP models. Furthermore, subgroup analysis of the primary randomized controlled trial showed a significant improvement in mortality from hypothermic sepsis (54% vs. 90%) in those treated with ibuprofen [90]. This presentation was associated with an exaggerated prostanoid response on admission to the study [76]; a suggestion that had laboratory-based testing been used to target therapy at a more immunologically homogeneous population, a beneficial signal could have been detected.

Pharmaceutical trial evidence targeting the inflammatory cascade in CI has been instructive largely through its failure. Novel immunomodulatory approaches aiming to reduce late-phase mortality in stratified patient populations have already demonstrated promise 17, 18, 75. Discarding NSAIDs – a relatively safe, cheap, and potentially efficacious class of drugs – based on limited data seems naïve. Further investigation may yet establish therapeutic eicosanoid manipulation as part of an immune-restorative arsenal in CI.

The administration of SPMs or their precursors, as either replacement for a lack of endogenous production for instance, from a failure to ‘class switch’ 64, 67, or as a supplement to host-generated mediators [20] in the face of an acute increase in catabolism [26], offers one strategy to combat failure of inflammatory resolution. Oral administration of eicosapentaenoic and docosahexaenoic acid have already been shown to increase plasma levels of SPMs to clinically relevant levels [77]. Alternatively, the use of drugs that act as resolution agonists may prove advantageous (Figure 3). In particular, there is compelling evidence that aspirin [42], statins [78], and potentially glucocorticoids [79] increase the generation of SPMs or their equally bioactive epimeric forms to augment aspects of innate immune function and promote the resolution of inflammation (Figure 1).

The concentrations of mediators achieved at the inflammatory site will be key to the efficacy of these therapeutic strategies. As Ricciotti and FitzGerald observe ‘it is one thing to show that putative pro-resolution products can be formed *in vitro* and that the synthetic compounds do exert pro-resolution actions when administered *in vivo* and another to document that the concentrations formed *in vivo* in the setting of inflammation are sufficient and necessary to mediate resolution’ [80]. Recent reports describing the isolation of high levels of SPMs in discrete human biological samples that follow predictable, modifiable, temporal profiles however partially allay these concerns 77, 81. Data suggesting SPMs may be exploited by pathogens in supraphysiological levels to induce a survival advantage (Box 4) indicates that attempts to exploit their therapeutic potential must not inadvertently induce a state of injurious resolution. Further caveats are discussed below.

Box 4 **Pathogen modulation of LM profiles**

The exploitation of LM properties by multiple pathogens underscores their biological potency and the potential pathogenicity of their dysregulation.

Both *Toxoplasma gondii* and *Pseudomonas aeruginosa* have been shown to utilize their independently generated 15-LOX variants on host substrate (AA) to create a microenvironment with supraphysiological concentrations of LXs, blocking DC activation and preventing their phagocytosis 93, 94. *Mycobacterium tuberculosis* (MTB) has been demonstrated to support replication and immune evasion via a similar yet distinct mechanism. Protective circulating levels of (anti-inflammatory) LXs appear to be generated via MTB stimulation of host biosynthesis (as opposed to via pathogen-generated enzymes). 5-LOX-deficient mice consequently displayed enhanced T helper (Th)1 responses and lower bacterial burden than wild type controls; a protective phenotype abolished by administration of a stable LXA_4_ analog [95]. Other authors have demonstrated the opposite of this effect – diminished Th1 responses (lower IL-12 and γ interferon) and consequent survival in tuberculosis-infected mice treated with MK 886 [96]. It seems likely that both early inhibition of pro-inflammatory LTB_4_ and subsequent excessive LXA_4_ may be pathogenic in this setting. This demonstration of the importance of both pro- and anti-inflammatory LOX products in mounting a successful defense against invading pathogens has recently been elegantly supported by work in zebrafish [97].

A further eicosanoid contributor to the pathogen–host interaction is PGE_2_. PGE_2_ production triggered by virulent but not avirulent MTB has been implicated as a pathogen-defense mechanism, with PGE synthase^−/−^ mice having significantly higher bacterial burden post-challenge as a result of a shift in macrophage fate profile [98]. In addition, the fungi *Candida albicans* and *Cryptococcus neoformans* secrete PGE_2_ [99] as a virulence mechanism [100]. In this context, PGE_2_-mediated inhibition of the transcription factor interferon regulatory factor (IRF)4, with resultant decrease in Th17 cell IL-17 production during cell differentiation was implicated. Blockade of host PGE_2_ in mice infected with *C. neoformans* led to increased survival [100].

The inhibition or antagonism of early-phase eicosanoids contributing to CIIID, the corollary and complementary strategy of ameliorating injurious resolution (Figure 2), may yet prove more tractable. As the above discussion has indicated, a growing body of evidence suggests that PGE_2_, PGD_2_, 15d-PGJ_2_, and LTB_4_ may be viable targets, whether through inhibition of enzymatic synthesis, enhancement of catabolism (e.g., via albumin), receptor antagonism, or monoclonal antibody therapy. Translational potential will be predicated upon description of both local and systemic eicosanoid profiles induced by severe inflammatory stress, establishment of robust and generalizable resolution indices in health and disease to gauge pathogenic dysfunction, and the trialing of pharmacological agents capable of selectively modifying PG and LT concentrations in relevant compartments.

Immunomodulatory drugs offer two clear advantages over current management of CIIID and subsequent HAI. The first is the ability to prevent infections prophylactically. Corrective therapy may be administered throughout ICU admissions to achieve a minimum baseline immune functional status, preventing new invasive pathogens from gaining traction. The second is their ability to support bacterial killing in an antibiotic-independent manner via augmenting innate immune function. Antibiotic resistance is one of the greatest challenges facing humans and poses a particular problem in the ICU patient cohort. Strategies to both therapeutically enhance pro-resolution activity [22] and reduce prostanoids-driven injurious resolution [82] have recently demonstrated preclinical success in lowering antibiotic requirements and/or increasing their efficacy. It is currently unknown if these will translate into the clinical setting.

## Concluding remarks

An excessive dysregulated inflammatory response is associated with adverse clinical outcomes in CI patients. Resolution-regulating lipid mediators contribute to this phenomenon being mechanistically involved in CIIID via both sustained production of early-phase prostanoids, (in particular PGE_2_) and reduced or insufficient late-phase SPMs.

Resolution therapies, rectifying LM profiles, are predicted to restore innate immune function, reducing susceptibility to HAI and its sequelae. To be proven clinically effective they will have to avoid the pitfalls that have befallen multiple pharmacological agents in CI, including previous trials of NSAIDs in sepsis (Box 3). Patients will have to be stratified and appropriate therapy delivered according to real-time assessments of inflammatory status (pro vs. anti), immune functional defects, and causative mechanisms. Therapy will ideally be targeted at specific biochemical pathways (e.g., PGE_2_-EP2/4 blockade as opposed to nonspecific COX-inhibition), and guided by quantifiable outcomes [17] in order to reduces off-target effects and minimize potential toxicity. Equally, a ‘magic bullet’ should not be expected. LM manipulation may form one part of multimodal immunomodulatory therapy, working synergistically with other agents. Finally, diverse demographic, clinical, and microbiological considerations may also modify treatment decisions. Although these stipulations ensure drug development will undoubtedly represent a substantial challenge, the chances of developing an effective therapy are significantly higher as a result. The potential clinical benefit suggested by observational data provides sufficient justification to pursue this strategy 83, 84, 85, 86, 87, 88.
